# Efficacy, safety and tolerability of artesunate-mefloquine in the treatment of uncomplicated *Plasmodium falciparum *malaria in four geographic zones of Nigeria

**DOI:** 10.1186/1475-2875-7-172

**Published:** 2008-09-09

**Authors:** Philip U Agomo, Martin M Meremikwu, Ismaila M Watila, Innocent J Omalu, Friday A Odey, Stephen Oguche, Valentine I Ezeiru, Olugbenga O Aina

**Affiliations:** 1 Malaria Unit, Department of Biochemistry and Nutrition, Nigerian Institute of Medical Research, PMB 2013, Yaba-Lagos, Nigeria; 2 Department of Paediatrics, University of Calabar, Calabar, Nigeria; 3 Department of Paediatrics, State Specialists Hospitals, PMB 1014, Maiduguri, Nigeria; 4 Department of Paediatrics, Faculty of Medical Sciences, University of Jos, Plateau State, Nigeria; 5 Regulatory Affairs Department, Oculus Pharmacare Limited, Lagos, Nigeria

## Abstract

**Background:**

The combination of artesunate and mefloquine has been reported to be effective against multi-drug resistant *Plasmodium falciparum *malaria, which has been reported in Nigeria. The objective of this multi-centre study was to evaluate the efficacy, safety and tolerability of the co-packaged formulation of artesunate and mefloquine in the treatment of uncomplicated malaria in two weight groups: those between 15 – 29 kg and ≥ 30 kg respectively.

**Methods:**

The trial was conducted in rural communities in the north-east, north-central, south-west and south-eastern parts of Nigeria. The WHO protocol for testing antimalarial drugs was followed. Outpatients having amongst other criteria, parasite density of ≥1,000 μl were enrolled. The co-packaged drugs were administered for 3 days at a dosage of artesunate, 4 mg/kg body wt/day and mefloquine, 25 mg/kg/body wt total) on days 0, 1 and 2. Patients were followed up for 28 days with the assessment of the parasitological parameters on days 1, 2, 3, 7, and 28.

**Results:**

Four hundred and forty-six (446) patients were enrolled and 431 completed the study. Cure rates in both treatment groups was >90% at day 28. The mean parasite clearance times in treatment groups I and II were 40.1 and 42.4 hours respectively. The combination of artesunate and mefloquine showed good gametocidal activity, (gametocyte clearance time of 42.0 & 45.6 hours in treatment groups I and II respectively). There were no serious adverse events. Other adverse events observed were headache, dizziness, vomiting and abdominal discomfort. There was no significant derangement in the haematological and biochemical parameters.

**Conclusion:**

This co-packaged formulation of artesunate + mefloquine (Artequin™) is highly efficacious, safe and well-tolerated. It is recommended for the treatment of uncomplicated *P. falciparum *malaria in Nigeria.

## Background

Malaria remains one of the greatest causes of morbidity and mortality in the world. Globally, there are between 300–500 million cases of clinical malaria every year, with 85% of these from Africa [1]. Currently, 1.5 to 2.7 million deaths are attributable to malaria annually, 90% of them in Africa [1]. In Nigeria, malaria is holoendemic hence clinical cases of the disease are seen throughout the year. It is the commonest cause of outpatient hospital attendance in all age-groups in the country [2]. Under five children are especially prone to develop the severe forms of the disease which, if not treated promptly can lead to death. Drug Therapeutic Efficacy Tests (DTET) conducted in different parts of Nigeria on chloroquine and sulphadoxine-pyrimethamine combination in 2002 showed adequate clinical and parasitological response (ACPR) of 39.2% and 56.7% respectively [3]. Thus, chloroquine and sulphadoxine-pyrimethamine are no longer efficacious in treating malaria in Nigeria [4,5]. The global malaria control strategy advocates prompt and adequate treatment with an effective antimalarial drug as an essential measure to reduce the morbidity and mortality arising from the disease [3]. In line with above findings, the Federal Ministry of Health considered a change in policy to artemisinin-based combination therapy (ACT), which has been shown to be effective in other countries. The rationale for the use of ACTs is to reduce the probability of resistance developing simultaneously to two drugs with independent mechanisms of action [5,6].

The artemisinin drugs are developed from the Chinese wormwood (*Artemisia annua*) and the derivatives, namely, artemether, artesunate and dihydroartemisinin have now gained popularity as short acting drugs which could be used in combination with drugs which have longer half-life [7,8]. Mefloquine has been reported to consistently show high treatment efficacy in African children [9,10] and in pregnant women [11]. This was in the era of antimalarial monotherapy. At that time, mefloquine, a 4-quinoline carbinol, was reported to be one of the most effective drugs in the treatment of malaria in Nigeria [12]. It was also found to be an effective suppressive prophylactic drug, when administered weekly or fortnightly against drug-resistant *Plasmodium falciparum *[13]. The successful treatment of falciparum malaria with regimens of artemisinin derivatives plus mefloquine has been reported in other countries [14-17]. The pharmacokinetics of mefloquine combined with artesunate in children with acute falciparum malaria in Thailand has also been studied [18]. Li *et al *[19] showed that artesunate has a broader stage-specificity of action than other antimalarial drugs. After oral artesunate, relative bioavailability of the drug was 82.0%. The parasite clearance time (PCT) and fever clearance time (FCT) were 6.5 hours and 24 hours respectively [20] and parasitaemia was reduced by 90% within 24 hours after starting treatment.

The rationale was based on the convincing evidence that a combination of two or more schizontocidal drugs will not only improve cure rate but could help reduce the rate of development of parasite resistance to either of the drugs in the combination. Thus, the combination of short-acting artemisinin derivative (artesunate) with longer acting mefloquine is expected to constitute a good ACT.

The Drug Therapeutic Efficacy tests (DTET) conducted on two such combinations, namely, artesunate + amodiaquine and artemether + lumefantrine in 2004 showed adequate clinical and parasitological response (ACPR) of 94.6% and 96.8% respectively [3]. The Federal ministry of health then changed the policy on malaria treatment to artemisinin-based combination therapy (ACT) [3].

However, there is the challenge of availability and affordability of ACTs. To improve better access to ACTs at affordable prices, Roll Back Malaria partners in the pharmaceutical industries were encouraged to pre-package ACTs, which could be used if found effective, approved and duly registered by the regulatory authorities. One such combination drug is Artequin™, a combination of artesunate and mefloquine, manufactured by MEPHA Ltd (Aesch, Basel, Switzerland). Although this combination has been reported to be efficacious elsewhere, there is need to determine the efficacy, safety and tolerability of this ACT among Nigerians.

This co-packaged formulation of artesunate and mefloquine has not been used before now in Nigeria. The outcome of therapeutic efficacy tests could be different in Nigeria, or even in different geographic zones of the country. It is, therefore, important to determine the efficacy, safety and tolerability of this co-packaged formulation of AM among Nigerian population. There is also the need to provide more options for malaria control in Nigeria.

The objectives of the study were:

• To evaluate therapeutic efficacy of a combination of artesunate plus mefloquine (AM) using the modified WHO seven-day *in vivo *test extended to 14 and 28 day follow-up period.

• To determine the safety and tolerability of AM in the treatment of acute uncomplicated *P. falciparum *malaria.

• To estimate gametocyte carriage and its reduction during treatment.

## Methods

### study design

This was a descriptive, open label, multi-centre, non-comparative trial of three-day regimen of a combination of AM for efficacy, safety and tolerability. Patients were stratified into two treatment groups according to their weights. Treatment group I consisted of those weighing between 15–29 kg, while treatment group II consisted of participants whose weight was ≥ 30 kg.

### Study sites

This multi-centre study was conducted in four geographical zones of the country. In southwest Nigeria, 2 health facilities in Ijede, Lagos were used for the study. Ijede is a rural community in Ikorodu Local Government Area, Lagos State. The second site was in Borno, north eastern Nigeria, where a Specialist Hospital, a General Hospital and University Teaching Hospital were recruitment points. The third site was the Primary Health Centre in Ikot Ansa, Calabar, south eastern Nigeria. The fourth site was located in north central Nigeria where ECWA Evangel Hospital, Jos University Teaching Hospital and Plateau State Specialist Hospital were enrolment points. All sites were considered to be homogenous and high malaria transmission areas, hence their suitability for trials of this nature.

### Inclusion criteria

Patients between 15–29 kg or ≥ 30 kg with mono-infection with a *P. falciparum *parasitaemia in the range of 1,000 to 250,000 asexual parasites per μl of blood, presence of axillary temperature ≥37.5°C and/or history of fever in the preceding 24 hours, informed consent by parent/guardian (in the case of children), ability to come for the stipulated follow-up visits, and easy access to the health facility.

### Exclusion criteria

Patients with danger signs such as: unable to drink or breastfeed, unable to sit or stand up, vomiting everything, recent history of convulsion, lethargic or unconsciousness were excluded. Others excluded were those with signs of complicated falciparum malaria, such as severe anaemia (PCV ≤ 15%), shock, bleeding disorders, coke colored urine, jaundice, presence of severe malnutrition by clinical examination and weight for height measurement, history of allergy to study drugs and pregnant women.

### Study procedures

Patients who met the enrolment criteria were recruited. Written informed consent was obtained prior to enrolment. Day 0 was the day of screening, clinical assessment, initial malaria smears, haematological and biochemical assessments and enrolment. Temperature was measured in the axilla using digital electronic thermometer. Venous blood was collected from enrolled patients for baseline laboratory indices. For biochemistry, liver enzymes (aspartate amino-transferase and alaninie amino-transferase), total and conjugated bilirubin and serum creatinine were done. Haematological parameters such as haemoglobin, white blood cells (WBC), and erythrocyte sedimentation rate were also investigated. The patients were allocated to either treatment group I (15 to 29 kg) or treatment group II (≥ 30 kg) and given the 3-day co-packaged drug at a dosage of artesunate (4 mg/kg body wt/day, total = 12 mg/kg) and mefloquine (total = 25 mg/kg body wt.).

The drugs were administered under supervision and patients were observed for 60 minutes. If vomiting occurred within 30 minutes of drug administration, the full dose was repeated. However, if it occurred 30–60 minutes, half the dosage was given again. Participants who vomited a second time were excluded from the study and referred for treatment with appropriate parenteral antimalarial regimen. Use of concomitant medications (including acetaminophen) were documented in the Case Report Form (CRF).

The patients returned on days 1 and 2 to complete the drug administration and for clinical assessment. They were also given appointment for days 3, 7, 14 and 28 for clinical examination and blood smears. They were also asked to return to the clinic on any other day should they have new complaints, or any change in their condition. Patients that failed to report at the health centre for the scheduled visit were followed to their residence by trial field workers.

### Discontinuation of treatment

Serious adverse events, loss of patient to follow-up, consent withdrawal or withdrawal as a result of treatment failure, were criteria for discontinuation of treatment.

### Efficacy assessments

Primary treatment efficacy was determined based on parastological cure rates on days 2, 3, 7, and 28 and by the times to parasite and fever clearance and from the proportion of patients without gametocytes. The other outcomes assessed were early treatment failure (ETF), late clinical failure (LCF) and late parasitological failure (LPF). Recrudescence denoted clinical recurrence of malaria after the initial clearance of parasite from circulation. Parasite reappearance after day was interpreted as either true recrudescence or a new infection. Thus, treatment efficacy for cure rates in our context were described as uncorrected since no DNA polymerase chain reaction (PCR) analysis was performed in any of the four sites.

### Safety assessments

All adverse events were monitored and recorded on the case report forms (CRFs). Haematological parameters, liver enzymes and creatinine were assessed for the purpose of detecting abnormal laboratory features that constitute adverse events. Efforts were made to assess patients that dropped out from the study for the 28 days of active follow up period for safety reasons.

### Sample size calculation

The sample size used for this drug trial was calculated from the table of anticipated proportion (WHO/HTM/RBM/2003) at 95% confidence level and 10% precision. Calculation was based on estimated cure rate for current artemisinin-based antimalarial drug treatment [3]. With this combination drug having anticipated proportion of treatment failure of less than 5%, the sample size for the trial drug should be 18 (EPI-INFO version 6.04). However, since a minimum sample size of 50 is recommended by the World Health Organization (WHO), between 50–55 patients were enrolled in each treatment group per site (100 to 110), to adjust for loss to follow up and withdrawals.

### Data analysis

Data generated from this trial were entered into EPI-INFO version 6.04. Microsoft excel software was also used to plot simple graphs. Various aspects of the data were subsequently analysed using SPSS statistical package version 11. Descriptive statistics were produced for different parameters before figures representing various observations were compared using X^2 ^or student t-test or analysis of variance (ANOVA) as appropriate. Pearson's correlation test was used to examine the relationship between selected variables.

### Parasite counts

At screening, thick and thin blood films were collected. Thick film was examined with a binocular microscope with an oil immersion objective lens to quantify the parasitaemia. Parasitaemia was measured by counting the number of asexual parasites against a number of leukocytes in the thick blood film, based on a putative count of 8,000 leukocytes per microlitre of blood or an adequate mean WBC in the population under investigation. The number of asexual parasites was counted against 200 leukocytes using a hand tally counter. The number of parasites per microlitre of blood was calculated by using the formula:

$$\text{Parasite Density }\left(\text{parasites }\mu {\text{l}}^{-\text{1}}\right)=\frac{\text{number of parasites }\times \text{ WBC count }\left(\text{8}000\right)*}{\text{Number of leukocytes counted }\left(\text{2}00\right)}$$

If *P. falciparum *gametocytes were seen, a gametocyte count was performed against 1000 leukocytes (WHO/MAL/82.988).

### Ethical issues

Written approval was obtained by the Ethics Review Boards (IRBs) of the various institutions for the study. The Heads of communities and authorities of the various health facilities also consented to the conduct of the study. The study was carried out in accordance with the principles laid down by the World Health Assembly of 1975 on Ethics in Human experimentation and the Helsinki Declaration. The study adhered to Good Clinical Practices (GCP) and conformed to the TDR Standard Operating Procedures.

Participants were informed of the aims, methods, anticipated benefits and potential hazards of the study. Written informed consent was obtained from each participant and/or parent/guardian of patients participating in the study. Subject were informed that they could withdraw consent to participate anytime without any consequence to them. This was written in English and the local dialects of the various communities.

## Results

### General

A total of 4,139 patients who had not taken any antimalarial medication in the previous seven days were screened in the four sites. Overall, 446 patients fulfilled the criteria for enrolment and were enrolled, but a total of 431 (96.6%) patients consisting of 203 in treatment group I (weight ≥ 30 kg) and 228 in treatment group II (weight 15 to 29 kg) completed the study. The demographic and clinical characteristics of the patients are shown in Table 1.

**Table 1:** Demographic and clinical characteristics of patients at enrolment (Day 0)

	**Group 1 Weight ≥30 kg**	**Group 2 Characteristics Child Weight 15–29 kg**
Sex ratio		
Male	78 (38.4%)	139 (61.0%)
Female	125 (61.6%)	89 (39.0%)
Mean age (Yrs)	22.5 ± 11.5	7.1 ± 2.7
Range	9–65	(3–13)
Mean weight (kg)	50.9 ± 14.1	20.5 ± 4.7
Range	30–94	15–29
Mean Parasite Density (μl^-1^)	19,797.6 ± 33,397.6	27,010.2 ± 35,704.4
Range	1000–220,000	1016 – 235,294
Geometric mean parasite density (μl^-1^)	6890.8	11,727.5
Mean axillary Temperature (°C)		
≥ 37.5°C	38.4 ± 0.75 (n = 94)	38.6 ± 0.79 (n = 132)

### Defaulters

**Figure 1:**
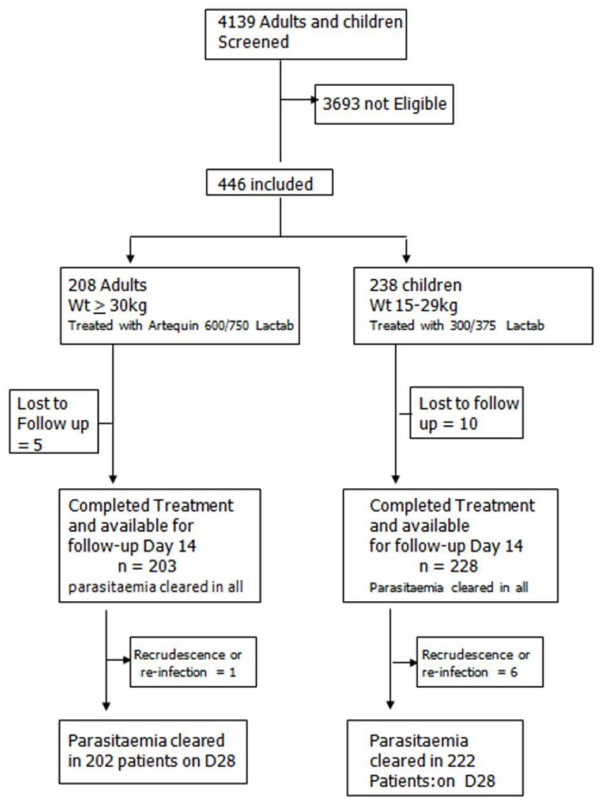
Artequin^® ^trial profile in four geographic zones of Nigeria.

A total of 15 patients defaulted as a result of withdrawal/loss to follow-up and/or protocol violation. The trial profile is shown in Figure 1.

### Efficacy of artesunate+mefloquine

#### The study outcome of trial participant is shown in table 2

**Table 2:** Study outcome of trial participants

**Characteristics/Outcome**	**Group I**	**Group II**	**Group I and II**
	**600/750 mg ≥30 kg**	**300/375 mg 15 – 30 kg**	**All Participants**
Number enrolled	208	238	446
Number completed	203	228	431
Withdrawal/loss to follow up	5(2.5%)	10(4.45)	15(3.5%)
Adequate Clinical and Parasitological Response (ACPR)			
Day 28*	202(99.5%)	222(97.4%)	424(97.45%)
Parasite Clearance Time	40.1 hrs	42.4 hrs	41.3

* Not Polymerase chain Reaction (PCR) corrected

##### Parasite clearance rate, profile and time

Results showed that on D1, 83(40.9%) of the 203 enrolled treatment group I (TG I) and 72 (31.6%) of the 228 enrolled treatment group II (TG II) no longer had any malaria parasites in their blood. The mean parasite densities in the remaining 120 in TG I and 156 in TG II were drastically reduced. The clearance rate dramatically increased to 92.1% and 91.7% for TG I and TG II respectively on D2 until total clearance was achieved in the remaining 16 in TG I and 19 in TG II on D3.

#### Treatment group I

In treatment group I, the geometric mean parasite density of the 203 participants enrolled on D0 was 6,890.8 parasites/μL of blood, which decreased to 30.74 parasites/μL on D1, giving a percentage rate of 99.6%. The rates on other days (i.e. using the geometric mean parasite densities) were as follows: D2 (99.98%), D3 (100%), D7 (100%) and D14 (100%). However, on day 28, one of those in TG I (in Lagos site) manifested low-grade parasite density of 360 parasite μL^-1 ^blood. It was not possible to ascertain whether this was recrudescence or of new infection. It was not possible to confirm recrudescence with polymerase chain reaction.

Parasite clearance time (PCT) in 203 participants in TG I was determined from the spread sheet data (WHO/MAL/82.988). Parasitaemia completely cleared in 83 patients within 24 hrs, 104 cleared in 48 hrs while 16 patients were cleared in 72 hours. Time to parasite clearance was calculated as follows:

$$\frac{\left(83\times 24)+(104\times 48)+(16\times 72\right)}{203}=\frac{8,136}{203}=40.1\text{hrs}$$

#### Treatment group II

In treatment group II, the geometric mean parasite density of the 228 enrolled patients on day 0 was 11,727.5 parasites/μ of blood which reduced to 54.2 parasites on D1, giving a percentage rate of 99.2% on D1 (Figure 1). The rates on the other days, using geometric mean parasite

density, were as follows: D2 (99.97%), D3 (100%), D7 (100%) and D14 (100%). As in TG I, six patients in TG II in three sites manifested low-grade parasitaemia on day 28. Time to parasite clearance (PCT) in 228 patients in TG II was also determined from the spread sheet. Here, parasitaemia had cleared in 72 patients within 24 hrs (D1), 137 participants within 48 hrs (D 2) and in the remaining 19 within 72 hrs (D3).

Time to parasite clearance was calculated as follows:

$$\frac{\left(72\times 24)+(137\times 48)+(19\times 72\right)}{228}=\frac{9672\text{hrs}}{228}=42.4\text{hrs}\text{.}$$

#### Temperature clearance profile

Result showed that 109 of the 203 enrolled patients in TGI had temperatures below 37.5°C. The mean temperature of the 94 in TG I patients with temperatures ≥ 37.5°C was 38.44 ± 0.75°C. The temperature dropped to a mean value of 36.4 ± 0.67°C in 24 hrs. The mean temperature of 132 patients in TG II who were febrile (T ≥ 37.5°C) was 38.56 ± 0.79°C. The temperature of these patients dropped to 36.57 ± 0.81°C by D1 (24 hrs). The fact that many patients were afebrile pre-treatment made it inappropriate to calculate fever clearance time.

#### Anti-gametocyte activity of artesunate+mefloquine

Three of the four sites investigated the gametocyte carriage rate and the changes in geometric mean gametocyte densities (GMGD) in the patients. The gametocyte carriage rate for TG I and TG II were 5.9% and 4.4% respectively. In ten patients where gametocytes were found, the mean gametocyte count dropped from pre-treatment levels on day 1. The gametocytes cleared in four patients in 24 hours, three were cleared in 48 hours and three in 72 hours. Time to gametocyte clearance in TG II was calculated (as in parasite clearance time) to be 45.6 hours.

In twelve TG I patients where gametocytes were found, the gametocytes cleared in five patients in 24 hours. It cleared in four patients in 48 hours and in the remaining three patients in 72 hours. The gametocyte clearance time in TG I was 42.0 hours.

#### Safety and tolerability

**Figure 2:**
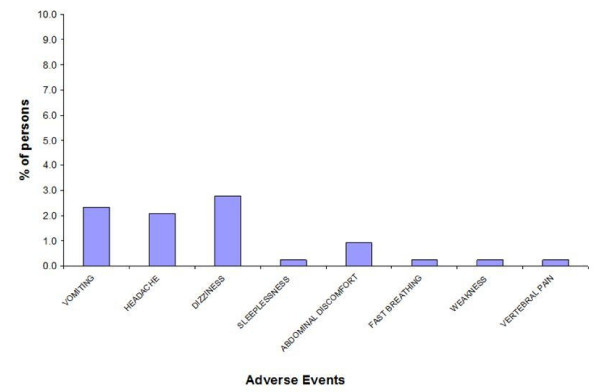
Adverse Events Reported By Study Participants.

No serious adverse event (SAE) was reported during the study in all the four trial sites. Many adverse events (AE) of the antimalaria drugs were most likely related to the underlying malaria disease. Of the 39 reports, 10 patients (2.3% of total patients treated) reported vomiting. The others were as follows: headache (9, 2.1%), dizziness (12, 2.8%) and abdominal discomfort (4, 0.9%). There was one report (0.2%) each of sleeplessness, fast breathing, weakness and back pain (figure 2.).

#### Laboratory indices of safety

The result of laboratory characteristics of patients at enrolment is shown in Table 3 and 4. The values in individual patients varied in accordance with the seriousness of the infection. However, the mean values of all parameters were within normal limits on D0. In table 3 the WBC count showed slight increase in both treatment groups. The increase was more prominent in the lymphocyte count with corresponding decrease in neutrophils as treatment progressed.

**Table 3:** Haematological Characteristics of Patients on Day 0, 7 and 28

	**Treatment Group 1 (≥30 kg)**			**Treatment Group 2(15–29 kg)**		
	**D0**	**D7**	**D28**	**D0**	**D7**	**D28**

Hb (mg/L)						
Male	11.67 ± 4.12	11.15 ± 2.37	12.19 ± 2.40	10.70 ± 2.65	9.53 ± 2.19	11.56 ± 1.34
Female	11.47 ± 2.49	10.27 ± 1.93	11.45 ± 1.42	10.84 ± 2.99	9.07 ± 3.20	11.58 ± 1.35
ESR	23.79 ± 26.74	14.34 ± 17.23	11.82 ± 14.96	42.73 ± 29.16	18.17 ± 23.66	11.53 ± 13.52
TWBC (× 10^9^/L)	5.27 ± 1.87	5.35 ± 1.93	5.15 ± 1.54	7.25 ± 1.11	5.91 ± 2.50	5.79 ± 4.85
Lymphocytes (× 10^9^/L)	2.22 ± 1.05	2.59 ± 1.09	2.66 ± 1.11	2.87 ± 3.34	2.97 ± 1.69	3.14 ± 3.08
Neutrophils (× 10^9^/L)	2.69 ± 1.28	2.55 ± 1.10	2.33 ± 0.80	4.10 ± 8.01	2.67 ± 1.21	2.47 ± 1.86
Monocytes (× 10^9^/L)	0.24 ± 0.16	0.09 ± 0.08	0.08 ± 0.08	0.09 ± 0.11	0.08 ± 0.08	0.08 ± 0.96
Eosinophils (× 10^9^/L)	0.12 ± 0.14	0.15 ± 0.15	0.09 ± 0.11	0.08 ± 0.11	0.15 ± 0.22	0.10 ± 0.11

**Table 4:** Biochemical Characteristics of Patients on Day 0, 7 and 28

	**Treatment Group 1 (> 30 kg)**			**Treatment Group 2 (15–29 kg)**		
	**D0**	**D7**	**D28**	**D0**	**D7**	**D28**

Alkaline Phosphatase (iu/L)	159.55 ± 121.58	156.95 ± 92.51	145.66 ± 99.40	220.64 ± 85.33	181.96 ± 69.98	197.12 ± 86.01
Aspartate amino Transferase (iu/L)	29.12 ± 19.54	21.96 ± 14.54	21.31 ± 16.15	38.58 ± 42.55	21.26 ± 14.21	24.47 ± 21.16
Alanine amino Transferase (iu/L)	20.41 ± 24.94	12.24 ± 10.0	14.23 ± 18.66	23.04 ± 20.40	10.64 ± 9.06	13.17 ± 9.99
Total biribin (μmoi/L)	6.16 ± 6.35	2.80 ± 4.61	4.53 ± 4.42	5.12 ± 5.63	0.95 ± 2.25	4.03 ± 3.59
Conjugated bilirubin (μmoi/L)	2.75 ± 3.25	0.29 ± 0.16	0.27 ± 0.19	0.49 ± 0.58	0.31 ± 0.16	0.38 ± 0.32
Urea	8.96 ± 7.14	8.58 ± 8.90	8.06 ± 8.02	8.22 ± 5.95	8.06 ± 7.43	8.43 ± 7.88
Creatinine	34.12 ± 31.81	38.12 ± 30.55	33.06 ± 91.10	21.97 ± 21.78	24.73 ± 20.92	22.16 ± 20.25

There was slight decrease in haemoglobin values on day 7 before returning to normal on day 28. All these changes were not statistically significant (p > 0.05). Other haematological parameters, viz, lymphocytes, neutrophils, monocytes and eosinophils also had varying values which were not statistically significant (P > 0.05) from the D0 values (Table 3).

The Biochemical values on D0 are also shown in Table 4. The mean starting values for total bilirubin was 6.16 ± 6.35 μmol^-l ^in TG I and 5.12 ± 5.63 μmol^-l ^in TG II. The level remained within this value from D0 to D28. There were also no significant decreases or increases (P > 0.05) in the values of serum alanine aminotransferase (ALT, GOT), serum aspartate amonitransferase (AST, GPT) and alkaline phosphotase (ALK).

## Discussion

The therapeutic efficacy study of co-packaged three day treatment preparation of artesunate and mefloquine in four different geographic areas of Nigeria showed that the combination is both effective and well tolerated in the treatment of acute uncomplicated falciparum malaria.

The geometric mean parasite density was drastically reduced in both treatment groups within 24 hours after treatment and completely cleared by day 3 in the remaining patients who completed the study. The parasitological response in both treatment groups were 100% on D3, D7, and D14 but 97.5% on day 28. The rapid clinical response was shown by a drop in temperature to normal values (viz. below 37.5°C) on the 2^nd ^day. This rapid clinical and parasitological response confirmed the previous findings of others [16-19,21,22] who have, for many years worked in countries where, as in Nigeria, multi-drug-resistant strains predominate.

Apart from the rapid clearance of asexual forms of *P. falciparum*, AM therapy was also beneficial in inducing significant reduction in gametocyte rate and density. The data suggest that AM ultimately cleared gametocytes from peripheral blood. This shows that AM exhibits a gametocidal effect. The second observation was that patients with mixed infections of *P. falciparum and Plasmodium malariae *were cured parasitologically and clinically.

It was observed in the course of this trial, that parasitaemia appeared on day 28 in one patient of the TG I and six patients of TG II. Parasitaemia was not associated with fever or other symptoms of malaria. Since analysis of the parasite genotypes was not performed, it was not possible to determine whether these were new infections or recrudescence. It is possible that the observed day 28 parasitaemia could be re-infections rather than recrudescence[21]. Falade *et al *[23], by genotyping new infections seen between 2^nd ^and 4^th ^week post-therapy, attributed this phenomenon to new infections. There is a need to perform more molecular genotyping of the parasite strains in subsequent trials to confirm this observation.

Based on the experience of this study, AM is safe and well-tolerated. The laboratory values were not significantly different pre-and post-treatment. The marginal variations in liver function test results may be related to stabilization of the liver following successful treatment. The same result was observed with mean haemoglobin values which returned to normal after recovery. The slight reduction in mean platelet count (data not presented) was consistent with the reported findings of relative thrombocytopenia in 50 to 75% of patients with acute malaria [24].

In conclusion, the results of this study have confirmed the efficacy of AM in the treatment of *P. falciparum *malaria in four sites in Nigeria. It also exhibited significant gametocidal activity. As observed by other workers, the rapid parasitological response corresponded to the fast clinical response.

## Competing interests

The authors declare that they have no competing interests.

## Authors' contributions

PUA, MMM, and IMW conceived the idea, wrote the protocol and supervised data collection and writing of this paper. PUA played a central role in coordinating the work of the 4 centres and is the contact author for this article. SO, FAO, IJO, VIE and OOA wrote and reviewed the paper before it was finally sent. VIE was a focal person for supply of the drugs. All authors made scientific contributions to this article.
